# Polydopamine-Functionalized Bacterial Cellulose as Hydrogel Scaffolds for Skin Tissue Engineering

**DOI:** 10.3390/gels9080656

**Published:** 2023-08-14

**Authors:** Kannan Badri Narayanan, Rakesh Bhaskar, Kuncham Sudhakar, Dong Hyun Nam, Sung Soo Han

**Affiliations:** 1 School of Chemical Engineering, Yeungnam University, 280 Daehak-Ro, Gyeongsan 38541, Gyeongbuk, Republic of Korea; indiaxenobiotic@gmail.com (R.B.); ksrsudhakar11@gmail.com (K.S.); ndh0826@gmail.com (D.H.N.); 2 Research Institute of Cell Culture, Yeungnam University, 280 Daehak-Ro, Gyeongsan 38541, Gyeongbuk, Republic of Korea

**Keywords:** bacterial cellulose, polydopamime, tissue engineering, murine fibroblasts, PrestoBlue, *Gluconacetobacter hansenii*

## Abstract

Bacterial cellulose (BC) is a natural polysaccharide polymer hydrogel produced sustainably by the strain *Gluconacetobacter hansenii* under static conditions. Due to their biocompatibility, easy functionalization, and necessary physicochemical and mechanical properties, BC nanocomposites are attracting interest in therapeutic applications. In this study, we functionalized BC hydrogel with polydopamine (PDA) without toxic crosslinkers and used it in skin tissue engineering. The BC nanofibers in the hydrogel had a thickness of 77.8 ± 20.3 nm, and they could be used to produce hydrophilic, adhesive, and cytocompatible composite biomaterials for skin tissue engineering applications using PDA. Characterization techniques, namely Fourier-transform infrared spectroscopy (FTIR), X-ray diffraction (XRD), field emission scanning electron microscopy (FE-SEM), and Raman spectroscopy, were performed to investigate the formation of polydopamine on the BC nanofibers. The XRD peaks for BC occur at 2θ = 14.65°, 16.69°, and 22.39°, which correspond to the planes of (100), (010), and (110) of cellulose type Iα. Raman spectroscopy confirmed the formation of PDA, as indicated by the presence of bands corresponding to the vibration of aromatic rings and aliphatic C–C and C–O stretching at 1336 and 1567 cm^−1^, respectively. FTIR confirmed the presence of peaks corresponding to PDA and BC in the BC/PDA hydrogel scaffolds at 3673, 3348, 2900, and 1052 cm^−1^, indicating the successful interaction of PDA with BC nanofibers, which was further corroborated by the SEM images. The tensile strength, swelling ratio, degradation, and surface wettability characteristics of the composite BC biomaterials were also investigated. The BC/PDA hydrogels with PDA-functionalized BC nanofibers demonstrated excellent tensile strength and water-wetting ability while maintaining the stability of the BC fibers. The enhanced cytocompatibility of the BC/PDA hydrogels was studied using the PrestoBlue assay. Culturing murine NIH/3T3 fibroblasts on BC/PDA hydrogels showed higher metabolic activity and enhanced proliferation. Additionally, it improved cell viability when using BC/PDA hydrogels. Thus, these BC/PDA composite biomaterials can be used as biocompatible natural alternatives to synthetic substitutes for skin tissue engineering and wound-dressing applications.

## 1. Introduction

Skin is the largest organ in the human body and plays a crucial role in protecting the organism from the external environment and maintaining homeostasis. It involves physiological functions, including sensory detection and thermal and hydration regulation, and helps fight against invading microorganisms via immune responses [1,2,3]. The skin comprises three layers: the outer layer (the epidermis), the middle layer (the dermis), and the bottom fatty layer (the hypodermis). Specialized cells and structures in the dermal and epidermal layers of the skin work together to perform all physiological activities [4]. The epidermis is the constantly regenerating 0.05–1.55 mm-thick layer, consisting of the outermost stratum corneum, followed by the stratum spinosum, stratum granulosum, and innermost stratum basale layers, making it a durable keratinized boundary [5]. The dermis ranges from 0.3 to 3.0 mm in thickness and provides nutritional and structural support to the epidermis. It contains dermal fibroblasts, which produce dermal structural components, including collagen, elastin, reticular fibers, and extracellular matrix (ECM) constituents such as proteoglycans, glycosaminoglycans, and several glycoproteins [6]. The innermost hypodermis comprises adipocytes that lie beneath the dermis and above the muscle. Each year in the United States, approximately 4.5 thousand patients undergo skin and wound care management using natural and synthetic materials for wound dressings [7]. Commercial skin care products, such as EZ-Derm, produced from porcine dermal collagen (https://ezderm.com/) (accessed on 13 June 2023); Biobrane, produced from porcine collagen bound to a silicon-nylon membrane (https://www.chelwest.nhs.uk/your-visit/patient-leaflets/burns/biobrane) (accessed on 13 June 2023); Integra^®^ dermal regeneration template (Integra template), produced using bovine collagen and glycosaminoglycan (GAG) on outer silicone membrane (https://www.integralife.com/file/general/1453795605-1.pdf) (accessed on 13 June 2023); and MatriDerm^®^, a unique collagen-elastin-template produced from elastin and collagens 1, 3, and 5 (https://matriderm.com/en) (accessed on 13 June 2023) are currently available as substitutes for the wounded human dermis, accelerating cell invasion, cell elongation, and proliferation.

The wound-healing process tightly controls the release of different growth factors, cytokines, and chemokines. Dermal fibroblasts secrete the constituents of ECM, collagen, and elastin, providing mechanical strength and elasticity to the skin [8]. These dermis-derived fibroblasts are a kind of functional mesenchymal stem cell (MSC), with remarkable potential as an alternative to MSCs for various therapeutic complications [9]. They play an important role in epithelial-mesenchymal transition (EMT) during wound healing and regeneration. They also secrete various growth factors and cytokines for epidermal proliferation, differentiation, and ECM formation [10]. Despite significant advancements in the understanding of the wound-healing processes at the cellular and molecular level, neither chronic wounds nor severe acute wounds have been successfully treated with a cost-effective, highly efficient revolutionary therapy [11,12]. To achieve this, fibroblasts need to be introduced into the wound site via skin tissue engineering to accelerate wound healing and skin regeneration processes for wound closure without scarring. However, the issue of employing fibroblasts in regenerative wound healing requires a highly cytocompatible and biocompatible biomaterial with good mechanical and cell adhesive properties as a tissue engineering construct, substituting the natural collagen, glycosaminoglycans (GAGs), and elastin properties to provide an extracellular matrix for the regeneration of cells for wound healing. Over the past few decades, there have been significant advancements in the fabrication of various biomaterials with different components that exhibit highly interactive properties with cellular components. Polymeric materials with highly compatible and adhesive cell-interacting surfaces with enhanced mechanical strength and flexibility can help cells proliferate for skin regeneration [13].

Natural ECM components such as collagen, fibronectin, laminin, elastin, and glycosaminoglycans can be used to fabricate scaffolds for skin tissue engineering. However, these biomaterials made from allogenic and xenogeneic sources have the potential to provoke an adaptive immune response and cytokine production, which could reduce their longevity [14]. Other natural biomaterials using chitosan, fibrin, silk, alginate, cellulose, glycogen, agarose, and dextran have also been used in skin tissue engineering [15,16,17]. However, among various natural polymers, cellulose is an abundant, sustainable source readily exploited for fabricating various biomaterials for tissue engineering applications [18]. Cellulose-based biomaterials can be derived from plant cellulose, bacterial cellulose, or cellulose derivatives. Compared to plant cellulose, bacterial cellulose (BC) is a three-dimensional network hydrogel matrix with monomers linked by covalent β-1,4-glycosidic linkages [19]. BC possesses unique characteristics such as a high crystallinity index (84–89%), uniform pore size distribution, high malleability, high hydrophilicity, and high water-holding capacity. It also has a high surface area-to-volume ratio, allowing strong interaction with other moieties via the high-density hydroxyl group on the surface. In addition, BC has the inherent properties of purity, biodegradability, biocompatibility, and nontoxicity, which make it an exceptionally versatile natural polymeric biomaterial for tissue engineering applications [20]. BC nanofibrils morphologically resemble ECM collagen fibrils, providing optimal interactions between proliferating mammalian cells and BC fibers [21]. However, the fundamental interaction between the components of BC and cells can be enhanced by functionalizing the surface of BC fibrils with various molecules. Polydopamine (PDA), the oxidized product of dopamine or other catecholamines, is structurally similar to the naturally occurring eumelanin [22]. Due to its extraordinary adhesive and antioxidant properties, PDA is used as a versatile coating material to cover the surface of many biomaterials [23]. In our study, we have developed a natural polymeric bacterial cellulose functionalized with PDA spheres as polymeric nanocomposite hydrogel scaffolds for enhanced adhesion and proliferation of mammalian cells for skin tissue engineering applications.

## 2. Results and Discussion

### 2.1. Characterization of BC and BC/PDA Hydrogels

**Figure 1:**
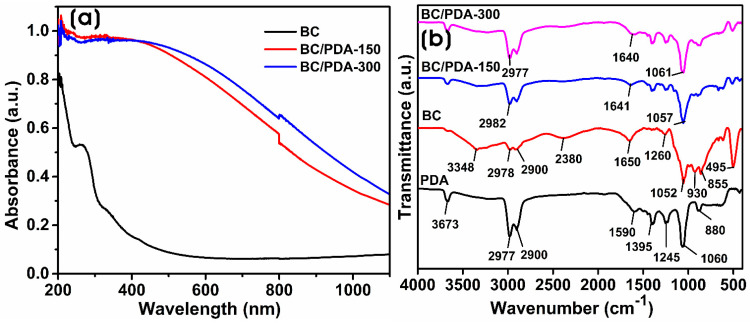
(**a**) UV-vis DRS and (**b**) ATR-FTIR of BC and BC/PDA hydrogels.

Generally, BC has no chromophores that absorb light in the ultraviolet-visible (UV-vis) spectrum. However, in our study, BC hydrogel showed an absorption peak at 260 nm, presumed to have come from the adherence of bacterial proteins or bacterial media components. The maximum absorbance of dopamine monomer lies at 280 nm [24]. On the other hand, BC/PDA-150 and BC/PDA-300 hydrogels containing polydopamine (PDA) change the biomaterial’s optical properties. PDA-functionalized BC hydrogels visually appear to have a light to dark gray or black color. The incorporation of PDA into BC nanofibers influences the optical absorption of the BC/PDA hydrogels on the visible and infrared spectra. Both BC/PDA hydrogels showed a broad band ranging from 1100 to 200 nm (Figure 1a). Jiang et al. [25] demonstrated that the size of incorporated PDA spheres alters the optical properties of biomaterials. The biocompatibility and adhesive properties of PDA can be used to coat various natural and synthetic polymeric substrates to modify them for various applications. Dopamine spontaneously oxidizes to form PDA micro- and nanospheres. The self-polymerization of dopamine happens via the oxidation of catechol to dopamine quinone in alkaline and aerobic conditions [26]. The hydroxyl groups of catechol and cellulose interact via hydrogen bonds, and the surface polarity of BC/PDA hydrogels becomes more hydrophilic with exposure to hydrophilic groups such as hydroxyl, carbonyl, and amino groups from PDA functionalization [27,28]. PDA-coated quartz showed a broad absorption band from 200 to 800 nm and a weak absorption peak at 290 nm on the UV spectrum [29].

The infrared spectra of BC, PDA, and BC/PDA hydrogels in the transmittance mode in the region between 4000 and 400 cm^−1^ are shown in Figure 1b. The BC spectrum showed typical bands of cellulose exhibiting a broad peak at 3348 cm^−1^ for O–H stretching vibration; the band at 2978 cm^−1^ corresponds to the C–H stretching for alkanes and CH_2_ asymmetric stretching [30], the peaks at 1052 cm^−1^ for C–OH stretching, 1260 cm^−1^ for the asymmetric stretching vibration of C–O–C, 1650 cm^−1^ corresponds to the H–O–H bending vibration of adsorbed water, and the peak at 2900 cm^−1^ appears due to the CH group [31,32]. Previous reports show that the absorption peaks of dopamine hydrochloride at 3500 and 3200 cm^−1^ are due to the stretching vibrations of O–H, N–H, and NH_2_, respectively [33]. In PDA, the absorption peak at 3673 cm^−1^ was attributed to the O–H stretching, and the sharp peaks between 3000 and 2800 cm^−1^ correspond to the N–H stretching. The peak at 1590 cm^−1^ corresponds to an overlap between N–H bending and conjugated C=C vibrations in the aromatic ring as found in indole or indoline derivatives [34,35]. Moreover, the small peaks at 1395 and 1245 cm^−1^ correspond to the CH_2_ bending vibration and stretching vibration of catechol hydroxyl C–O and C–N, respectively [36]. In BC/PDA hydrogels, the PDA peaks predominate, showing that PDA covers the surface of the BC’s nanofibrous structure, and the intensity of PDA peaks is higher with BC/PDA-300, indicating higher amounts of PDA on the BC surface.

**Figure 2:**
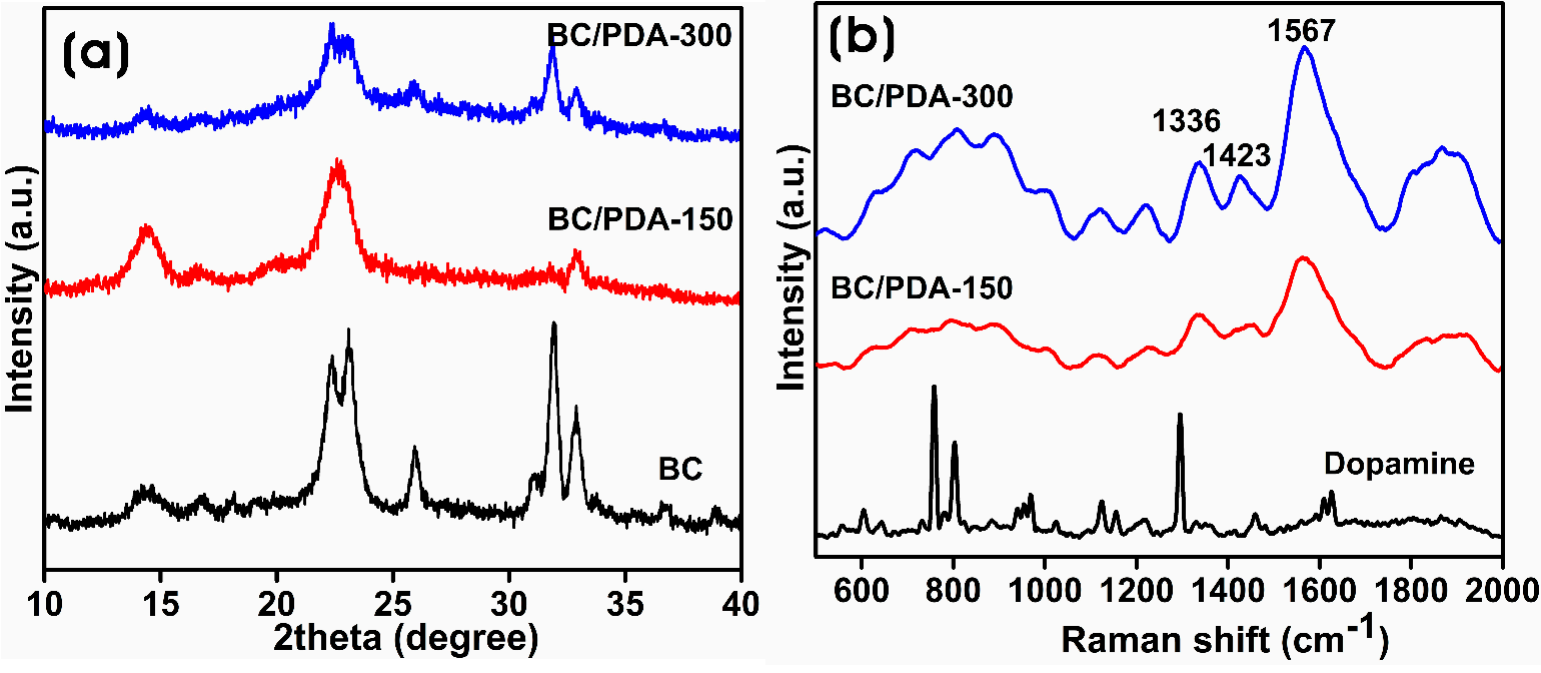
(**a**) X-ray diffraction patterns of BC and BC/PDA hydrogels, and (**b**) Raman spectra of dopamine and BC/PDA hydrogels.

X-ray diffraction (XRD) yields information about the crystallite size and crystallinity of biomaterials. Luo et al. [37] demonstrated that PDA exhibits a broad, amorphous peak at 23.2° with no crystalline reflections. Figure 2a shows the XRD pattern of the BC and BC/PDA hydrogels, showing the presence of crystalline reflections. The diffraction peaks for the synthesized BC at 2θ = 14.65°, 16.69°, and 22.39° were attributed to the planes of (100), (010), and (110) of cellulose type Iα [38]. The peaks at 25.97°, 31.94°, and 32.89° were related to impurities in the culture media, which could be removed via purification procedures [39]. The crystallite sizes of BC, BC/PDA-150, and -300 were 5.5, 5.3, and 5.4 nm, respectively, whereas the degree of crystallinity was 95.62, 84.94, and 65.11%. Jia et al. [40] reported the crystallite size of BC at 5.6 nm, which decreased with the integration of chitosan hydrochloride. Bacterial cellulose produced by *Acetobacter xylinum* showed high crystallinity in hydrogels. In another instance, BC produced by *A. xylinum* exhibited higher crystallinity (93%), with a crystal size of 5.2 nm [41]. Betlej et al. [42] demonstrated the production of BC using a sweet potato medium made of plant components, which showed a lower crystallinity of 27%, whereas BC obtained from a Hestrin–Schramm substrate showed 65% crystallinity. Compared to the crystallinity of BC/PDA-150, there was a decrease in the crystallinity of BC/PDA-300, which displayed a surface functionalization of BC nanofibers with an amorphous PDA structure, which had a significant impact on the crystallinity of the BC/PDA hydrogels.

PDA is produced via the oxidative polymerization of dopamine monomers under alkaline conditions and functionalizes BC nanofibers at room temperature. The PDA structure consists of many benzene rings with catechol and quinone functional groups of dopamine. Figure 2b shows the Raman spectra of dopamine, BC, and BC/PDA hydrogels. A ×100 objective lens was used to collect the spectra with a 532 nm laser and 1800 g/mm. The Raman spectrum of dopamine showed characteristic bands at 758, 948, 1326, and 1415 cm^−1^ attributed to C–C stretching, and the peaks at 1294 and 800 cm^−1^ arise due to the C–O and C–N stretching, respectively. Both BC/PDA-150 and -300 show two broad peaks at 1336 and 1567 cm^−1^, coming from the vibration of aromatic rings and aliphatic C–C and C–O stretching, respectively, which confirms the formation of PDA on the surface of the BC nanofibers of the hydrogel [25]. BC/PDA-300 with higher-intensity peaks of the catechol group indicating the increased catechol content of the polymerized PDA from the higher dopamine concentration [43].

**Figure 3:**
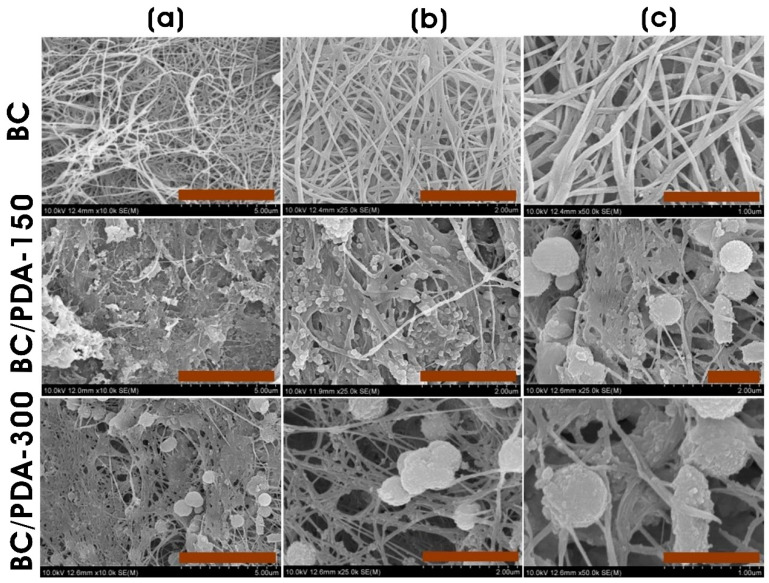
FE-SEM of BC and BC/PDA hydrogels at different magnifications. Scale bar: (**a**) 5 µm, (**b**) 2 µm, and (**c**) 1 µm.

Figure 3 shows the field emission scanning electron microscopy (FE-SEM) images of the surface morphology of BC and BC/PDA hydrogels. The BC hydrogel exhibited a three-dimensional (3D) nanofibrous network structure. The BC matrix consists of randomly arranged cellulose fibrils with a 77.8 ± 20.3 nm diameter and void spaces between them. *G. hansaneii* produces cellulose nanofibrils utilizing glucose moieties by conjugating them via β-(1 → 4) glycosidic linkages and stabilizing them using intramolecular hydrogen bonding, forming a reticulated structure [44]. In BC/PDA hydrogels, PDA can strongly interact with BC nanofibers with highly reactive functional moieties such as amine, imine, and catechol, enhancing the interaction with biomolecules for tissue engineering applications [45]. The surface of BC nanofibers in hydrogels is changed by functionalization with PDA, and there is a thin layer of PDA coating on the entire surface of the BC nanofibers along with rougher surfaces. Along with these, there are also appearances of very fine to larger PDA spheres immobilized on the BC fibers. BC/PDA-150 shows the adherence of PDA spheres with a diameter of 0.25 ± 0.18 µm, whereas BC/PDA-300 has 0.65 ± 0.14 µm PDA spheres. The treatment of BC hydrogels with an increased dopamine concentration resulted in the formation of larger PDA spheres in BC/PDA-300.

**Figure 4:**
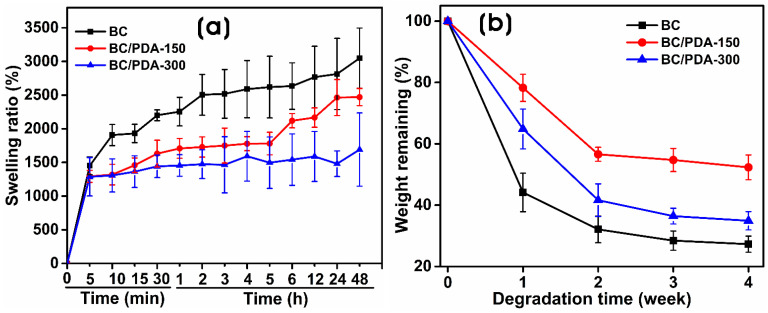
(**a**) Swelling ratios and (**b**) in vitro degradation studies of BC and BC/PDA hydrogels from 0 to 48 h.

Figure 4a shows the swelling ratio of BC and BC/PDA hydrogels via gravimetric assay. The swelling ratio indicates the water retention capacity of these hydrogels. These naturally crosslinked, nontoxic, covalently bonded BC hydrogels have numerous hydrophilic functional groups that interact with water molecules by retaining them [46]. With the increased functionalization of BC nanofibers with PDA, there was a dramatic decrease in the swelling ratios of the BC/PDA hydrogels. Within 5 min, hydrogels of BC, BC/PDA-150, and -300 reached swelling ratios of 1451.23 ± 132.7, 1293.75 ± 89.3, and 1287.23 ± 285.42, respectively, whereas, after 48 h, they became saturated with swelling ratios of 3049.16 ± 446.01, 2470 ± 127.4, and 1691.14 ± 544.02. BC has a good affinity for water and swells in water due to the hydroxyl and amino groups; however, aromatic and quinone structures are formed during the oxidation process of dopamine [47]. When BC is functionalized with PDA, it decreases the number of free hydrophilic groups and reduces the swelling ratio.

Swelling ratio is an extrinsic property that depends on the hydrogel’s surface area-to-volume ratio. This is feasible due to the hydrophilic and elastic properties of the polymeric network, which enable water absorption with subsequent increases in volume and create elastic tension within the polymeric network. Moreover, the hydroxyl groups of catechol and the amino groups of PDA strongly cross-network with the BC nanofibrils. Also, the PDA microspheres with larger sizes on BC/PDA have a lower swelling rate than the surrounding BC gel, resulting in an overall lower swelling ratio for BC/PDA [48,49]. Similar results were reported by Kacvinska et al. [50], who found that PDA coating of an implantable bilayer scaffold of collagen and chitosan (Coll/Chit) or collagen and a calcium salt of oxidized cellulose (Coll/CaOC) over electrospun gelatin, polycaprolactone, and CaOC affected swelling capacity and porosity.

Generally, the in vivo degradation of biomaterials occurs primarily via the enzymatic activity of biological systems and the hydrolytic breaking of polymeric backbones and crosslinkers, resulting in the structural breakdown of the hydrogels. The in vivo degradation rate of the hydrogel is also influenced by various factors such as pH, temperature, the composition of the material, and exposure time [51,52]. To understand the degradation behaviors of the BC and BC/PDA hydrogels under biological circumstances that mimic in vivo conditions, hydrogels were incubated at 37 °C under shaking at 50 rpm in sterile PBS (pH 7.4) solution. As expected, the degradation of BC was higher than that of the BC/PDA hydrogels. BC showed degradation up to 72.7 ± 2.6% after 28 days, whereas BC/PDA-150 and -300 showed 47.7 ± 4.0% and 65.0 ± 2.9%, respectively (Figure 4b). The degradation rate is also affected by hydrophilicity, porosity, crosslinking, functionalization, and the interaction and adsorption of biomolecules. In BC/PDA-300, the excess PDA spheres on BC nanofibers degraded with time compared to BC/PDA-150, leading to further weight loss of the hydrogel. Chen et al. [53] showed that with degradation time, the size of the PDA spheres decreases, and their morphology changes to nanosheets.

Moreover, the degradation products of PDA suppress the inflammation of macrophages via downregulation of the TLR-4-MYD88-NFκB pathway [54]. Thus, experimental results suggest that PDA-functionalized BC biomaterials can be employed in wound dressing and tissue engineering applications. They can easily integrate with a biological system with many therapeutic benefits and can be easily removed without releasing harmful degradation byproducts [55,56,57].

**Figure 5:**
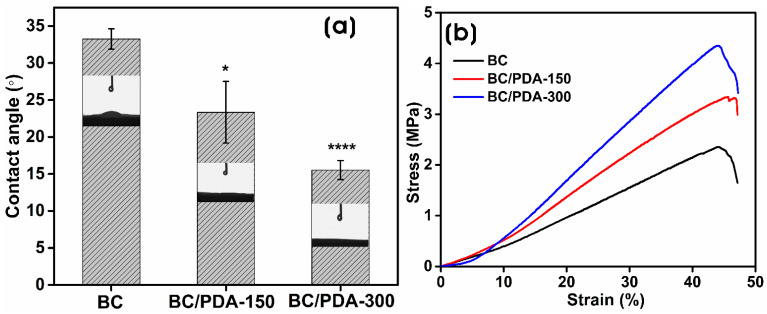
(**a**) Water contact angle measurements and (**b**) tensile stress–strain curves of the BC and BC/PDA hydrogels. The statistical significance between BC and BC/PDA was calculated using an unpaired *t*-test with Welch’s correction (* *p* ≤ 0.05, **** *p* < 0.0001).

Surface wettability is the contact angle of a water droplet on a solid surface, mainly influenced by the coating material and the roughness average of the surface (Ra). The surface wettability of BC and BC/PDA hydrogels was measured via the sessile drop method. Figure 5a shows the water contact angle measurements of BC and BC/PDA hydrogels. The contact angle of BC hydrogels was 33.3° ± 1.4°, and BC functionalized with PDA, i.e., BC/PDA-150 and -300, were 23.3° ± 4.2° and 15.5° ± 1.3°, respectively. With the increase in the concentration of PDA on the BC hydrogel, there was an increased functionalization of BC nanofibers, causing a significant decrease in the contact angle. The reduction in contact angle is due to the presence of more exposed hydrophilic groups on the surface of the PDA-functionalized BC nanofibers of the hydrogel. These findings are consistent with previous reports where PDA was used to functionalize various surfaces [58,59,60]. Sanbhal et al. [61] reported a reduction in the water contact angle of the PDA-coated hydrophobic surface of polypropylene fibers up to 74.1°. The functionalization of BC nanofibers with PDA enhances surface hydrophilicity, which is suitable for the adherence of cells and the adsorption of proteins and other biocomponents for cell proliferation in the aqueous environment. Moreover, the hydroxyl groups of catechol and the amino groups of PDA cross-network the BC nanofibrils. PDA microspheres with larger sizes have a lesser swelling rate than the surrounding BC gel, which can cause internal cracks and as well as different roughness and topography on the BC/PDA surface than the BC surface [48]. Yang et al. [62] demonstrated that increasing PDA content on BC composites creates a non-observable water contact angle compared to BC.

The mechanical properties of BC and BC/PDA hydrogels are presented in Figure 5b. The stress–strain curves of hydrogels show 1.64 ± 0.63 MPa of tensile stress and 44.9 ± 3.1% of tensile strain for BC hydrogels, whereas BC/PDA-150 and -300 showed tensile stresses of 2.77 ± 0.49 and 3.9 ± 0.55 MPa, respectively, and tensile strains of 47.68 ± 4.2 and 42.9 ± 1.82%. The enhanced mechanical properties of BC/PDA hydrogels compared to unfunctionalized BC hydrogels clearly indicate the role of PDA functionalization of BC nanofibers. The enhancement may be attributed to the interfacial interactions of BC nanofibers with PDA molecules. Kang et al. [63] demonstrated that the strong adhesive force of PDA enhances the tensile strength of the composite fibers of graphene oxide and polyvinyl alcohol matrix via a strong hydrogen bond.

### 2.2. In Vitro Cytocompatibility Assay

**Figure 6:**
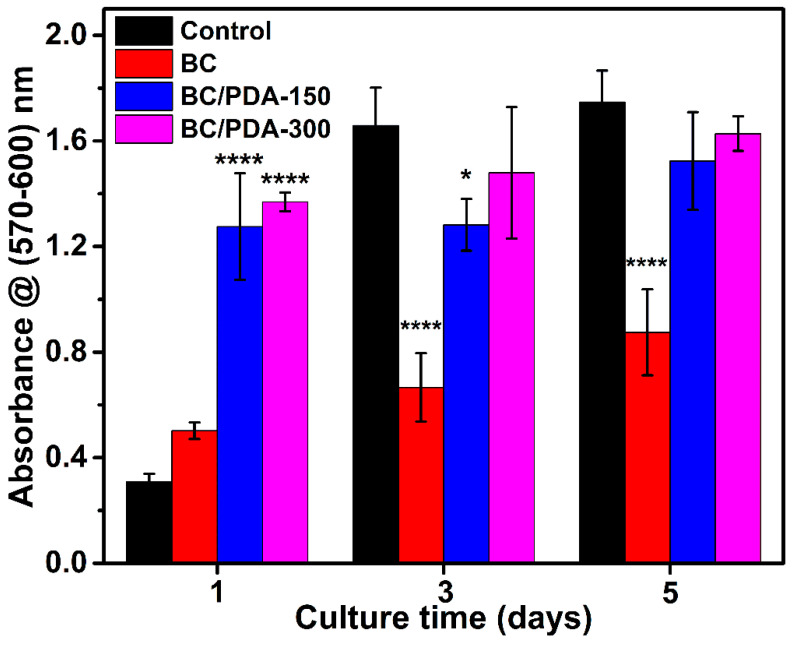
In vitro cytocompatibility of BC and BC/PDA hydrogels. The statistical significance between hydrogels and control was calculated using an unpaired *t*-test with Welch’s correction (* *p* ≤ 0.05, **** *p* < 0.0001).

Cell adhesion and proliferation are crucial indicators of a biomaterial’s cytocompatibility. BC hydrogels with nanofibers are naturally nontoxic and cytocompatible; however, to evaluate the effects of functionalization of BC with various concentrations of PDA on cell processes, murine NIH/3T3 fibroblasts were cultured in vitro. The metabolic activity of cultured murine fibroblasts for up to 5 days is shown in Figure 6. The metabolic activity indicates the cell viability of the biomaterial hydrogel and evaluates the proliferation of fibroblasts using a resorufin-based PrestoBlue assay. The assay measures viable cells’ metabolic ability to reduce resazurin to resorufin enzymatically. The reduction of resazurin or the generation of resorufin is directly proportional to the number of metabolically active viable cells, which can be quantified colorimetrically [64]. On day 1, BC/PDA-150 and -300 showed significantly higher metabolic activity than the control wells. This is presumed to happen due to the strong adhesion of cells to the surface of PDA on BC/PDA hydrogels and subsequent cell survival and proliferation. However, there was a saturation in the metabolic activity of fibroblasts in BC/PDA hydrogel scaffolds due to the insufficient migration and proliferation of cells into the hydrogel pores on days 3 and 5.

In addition, BC hydrogels with cultured fibroblasts showed lower metabolic activity on all days, possibly due to the improper adhesion of cells on the BC fibers compared to BC/PDA hydrogels, leading to less proliferation. However, a progressive increase in the metabolic activity on BC hydrogels indicates that the adhered and surviving cells improve the condition on the BC surface for the further attachment and migration of proliferating cells. Overall, PDA functionalization of BC hydrogels significantly increased the adhesion and proliferation of cells for tissue engineering applications.

**Figure 7:**
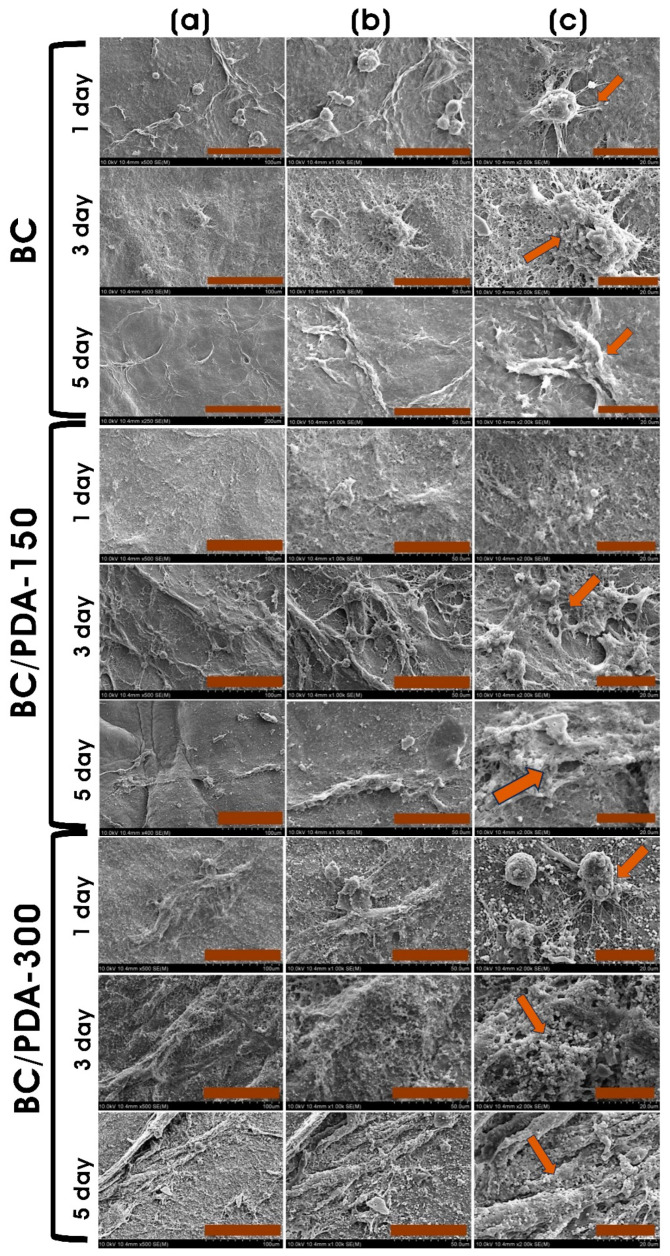
FE-SEM images of BC and BC/PDA hydrogels with cultured murine embryonic NIH/3T3 fibroblasts on different days. Scale bar: (**a**) 100 µm, (**b**) 50 µm, and (**c**) 20 µm. Arrows indicate cell morphology.

Polydopamine is an excellent adhesive material with superhydrophilic properties. Functionalizing BC with PDA brings hydrophilicity to the biomaterial and subsequent adherence of mammalian cells [65]. On all BC and BC/PDA surfaces, murine fibroblasts adhere and display a stretched and elongated morphology (Figure 7). PDA promotes more cell attachment by promoting cell–matrix adhesion, and the formation of cytoplasmic protrusions (filopodia) is visible. With the increase in culture days, apparent structures indicating a high density of filopodia were observed, with cell–matrix and cell–cell interactions. In all hydrogels, on the first culture day, the ball-like morphology of filopodia endings was clearly observed; however, with increasing culture days, flattened filopodia shapes were observed [66]. Moreover, with an increase in the PDA concentration on the BC/PDA surface, there was an evident indication of more layers of cells than BC surfaces (Figure 7). The higher number of cells in confluency could have arisen from the efficient adhesion and proliferation caused by the diffusion of nutrients through the pores of different layers of scaffolds [50].

## 3. Conclusions

Our study prepared bacterial cellulose-functionalized polydopamine (BC/PDA) hydrogels as composite biomaterials via impregnation in an aerated alkaline dopamine solution without crosslinkers. The formation of PDA on the surface of BC nanofibers without disturbing the stability of BC fibers was confirmed via FTIR and Raman spectroscopy. The surface wetting ability and mechanical strength increased with the PDA functionalization of BC hydrogels. The swelling ability of the BC/PDA hydrogels decreased with an increase in PDA content. The BC composite hydrogel with PDA closely mimicked the extracellular matrix of skin tissue in morphology. These composite hydrogels also enabled the adhesion of cells and the diffusion of nutrients across the biomaterials for cell survival, proliferation, and migration. The functionalization of BC with PDA enhanced the cytocompatibility of the biomaterial for skin tissue engineering. Murine fibroblasts on BC/PDA hydrogels increased cell survival while exhibiting more significant metabolic activity and enhanced proliferation. Overall, the in vitro investigation has proven the biocompatible and nontoxic nature of BC/PDA with good adhesion and proliferation of murine fibroblasts. However, more in vivo studies are required for complete validation of these biomaterials, and it can be concluded that these polydopamine-functionalized natural polymeric hydrogels can be an effective candidate for tissue regeneration and wound dressing applications.

## 4. Experimental

### 4.1. Materials

*Gluconacetobacter hansenii* (ATCC 23769) was purchased from the American Type Culture Collection (ATCC, Manassas, VA, USA). Mannitol, yeast extract, and peptone were purchased from MB Cell (Republic of Korea). Glucose, sodium hydroxide (NaOH), ammonia water (extra pure, assay 25–29%), and ethyl alcohol were purchased from Duksan Pure Chemicals (Gyeonggi-do, Republic of Korea). Dopamine hydrochloride was obtained from Sigma-Aldrich (St. Louis, MO, USA). Eagle’s minimum essential medium (EMEM), Dulbecco’s phosphate-buffered saline (DPBS), fetal bovine serum (FBS), penicillin/streptomycin (10,000 U/mL), and 0.25% trypsin-EDTA were purchased from Gibco^TM^ (Thermo Fisher Scientific, Waltham, MA, USA). Invitrogen^TM^ PrestoBlue^®^ cell viability reagent was purchased from Thermo Fisher Scientific (Waltham, MA, USA).

### 4.2. Preparation of Bacterial Cellulose (BC) Hydrogels

Bacterial cellulose (BC) hydrogel scaffolds were prepared using a culture of *G. hansenii*. Briefly, the culture of *G. hansenii* was statistically cultivated in 100 mL of mannitol broth containing 25 g/L mannitol, 5 g/L yeast extract, and 3 g/L peptone in a 250 mL conical flask for 7 days at 28 °C. After incubation, the BC scaffolds were added to 0.5% NaOH and autoclaved at 121 °C for 15 min, then washed with deionized water, freeze-dried, and stored at 4 °C.

### 4.3. Preparation of Polydopamine-Functionalized BC (BC/PDA) Hydrogels

A 10 g quantity of freeze-dried BC scaffold was placed in a petri dish, immersed in a 100 mL water/ethanol (60:40) solution containing 500 µL of ammonia water, and placed in a shaking incubator at 50 rpm at 40 °C for 1 h. To this mixture, 0.15 and 0.3 g of dopamine hydrochloride solution were added dropwise, incubated for 3 days, and designated as BC/PDA-150 and -300, respectively. These treated BC hydrogels were taken and washed three times in DPBS, freeze-dried at −80 °C overnight, and stored at 4 °C for characterization and in vitro cell culture experiments.

### 4.4. Characterization of BC and BC/PDA Hydrogels

The incorporation of PDA with BC scaffolds was analyzed via an ultraviolet-visible diffuse reflection spectroscopy (UV-vis DRS) spectrophotometer on a Varian Cary 5000 instrument (Agilent Technologies, Santa Clara, CA, USA). The crystallite size and crystallinity index of the BC/PDA hydrogels were determined using the thin film X-ray diffraction (XRD) technique with a PANalytical X’Pert PRO MRD (Almelo, The Netherlands). The data were recorded in the range of 10–90° using a CuK source (λ = 1.5418 Å) operating at 40 kV and 30 mA. The crystallite size of the cellulose produced was calculated using the Scherrer equation: *D*_110_ = κλ/β cosθ, where κ is the Scherrer constant, λ is the X-ray wavelength, β is the full-width half maximum (FWHM) of the (110) peak, and θ is the Bragg angle [67]. The crystallinity of the BC and BC/PDA hydrogels was also calculated using the peak deconvolution method [68]. The relative crystallinity (%) was calculated as follows: crystalline area/(crystalline + amorphous) area in the range 10–40°. The interactions of BC and PDA functional groups in BC/PDA were analyzed using attenuated total reflectance-Fourier transform infrared spectroscopy (ATR-FTIR) with Spectrum 100 (PerkinElmer, Waltham, MA, USA). The spectra were recorded by accumulating 64 scans in the 400–4000 cm^−1^ range at a 4 cm^−1^ resolution. Field emission scanning electron microscopy (FE-SEM) was used to analyze the surface morphology of BC/PDA scaffolds using the instrument S-4800 (Hitachi, Kyoto, Japan). The surface functionalization of BC nanofibers with produced polydopamine was characterized by Raman spectroscopy (XploRA^TM^ Plus; Horiba Scientific, Chiyoda-ku, Tokyo, Japan) to examine the presence of aromatic rings in PDA with Labspec 6 spectroscopy suite software.

#### 4.4.1. Mechanical Properties

The mechanical properties of BC and BC/PDA hydrogel scaffolds were evaluated via tensile testing using a tabletop universal tester MCT-1150 (A&D Company, Ltd., Tokyo, Japan) equipped with a 500 N load cell and a 10 mm/min tensile rate. The tests were performed in a wet state at room temperature (~25 °C) after immersing the rectangular hydrogel scaffolds (length × breadth; 20 mm × 10 mm) for 24 h in deionized water, and the excess water was removed with filter paper. At least five replicates were tested under a given condition.

#### 4.4.2. Surface Wettability

To quantify the surface polarity, contact angle measurements were realized at 25 °C with the OCA-20 contact angle instrument (DataPhysics Instruments, Filderstadt, Germany) using a sessile drop technique over the BC and BC/PDA hydrogels [69]. The static water contact angle (θ) with freeze-dried hydrogels was measured by dropping 10 µL of deionized water using an automatic dispensing system on the surface of the hydrogel, and the contact angle data was taken after 2 s using SCA20 software.

#### 4.4.3. Swelling Ratio

The swelling ratios (SR) of BC and BC/PDA hydrogels were evaluated via the gravimetric method. The freeze-dried hydrogels (W_i_) (diameter × height; 8 mm × 1 mm) were weighed and immersed in a tube containing 10 mL of phosphate-buffered saline (PBS; pH 7.4) and incubated at 37 °C and 50 rpm in an orbital shaker. At different time intervals, the swollen hydrogels (W_t_) were taken, and the excess solution was removed via gentle tapping with filter paper and weighed. The SR (%) was calculated as follows: SR (%) = [(W_t_ − W_i_)/W_i_] × 100, where W_t_ and W_i_ are the weights of the swollen hydrogels at a particular time and initial time, respectively [70].

#### 4.4.4. In Vitro Degradation

The freeze-dried BC and BC/PDA hydrogels (diameter × height; 8 mm × 1 mm) were initially weighed and immersed in 10 mL of sterile phosphate-buffered saline (PBS; pH 7.4) and placed on an orbital shaker at 50 rpm at 37 °C for 28 days [71]. Every 7 days, the buffer solution was aspirated and washed. Then, the hydrogels were freeze-dried, and the weight of the hydrogels was recorded. The remaining weight percentage at different intervals was expressed as the percentage of the ratio of the remaining weight of the hydrogel to the initial weights. Four samples were prepared at each time point, and each experiment was repeated three times.

### 4.5. In Vitro Cytocompatibility Evaluation of Hydrogels

#### 4.5.1. Adherent Cell Culture

Adherent murine NIH/3T3 fibroblasts were cultured in a 75 cm^2^ culture flask with complete EMEM medium containing 10% FBS and 1X penicillin/streptomycin, and the medium was changed every 48 h. When cells were 80% confluent, they were trypsinized with 1X trypsin-EDTA at 37 °C for 3 min, and the dissociated cells were centrifuged at 1000 rpm for 5 min. The pelleted fibroblasts were resuspended in a fresh, complete EMEM medium and stained with trypan blue, and viable cells were counted with a hemocytometer.

#### 4.5.2. PrestoBlue Assay

In vitro cytocompatibility of the BC and BC/PDA hydrogels was assessed using NIH/3T3 fibroblasts, similar to that previously reported [72]. BC and BC/PDA hydrogels (11 mm diameter) were sterilized in a graded ethanol series, washed in DPBS, and immersed in EMEM medium overnight before being placed in a 24-well tissue culture plasma-treated plate. On the surface of these hydrogels, approximately 5000 cells were seeded and incubated in a complete EMEM medium containing 10% FBS and 1% antibiotics. At different incubation periods, the metabolic activity of the proliferating fibroblasts was evaluated via the PrestoBlue assay. Cells cultured on 2D polystyrene dish wells were considered controls.

For visualization of cultured fibroblasts on different hydrogels, the hydrogels with grown fibroblasts at different time periods were fixed with glutaraldehyde and freeze-dried after dehydration in an ethanol series [73]. The freeze-dried samples were placed on aluminum stubs and coated with platinum using an ion sputter coater (Model: MC1000; Hitachi Ltd., Tokyo, Japan), and the morphology of fibroblasts on hydrogels was imaged using FE-SEM.

### 4.6. Statistical Analysis

Experiments were performed three times, and the data were expressed as means ± SD of three independent replicates unless otherwise specified. The statistical significance between hydrogel and control was calculated using an unpaired *t*-test with Welch’s correction (* *p* ≤ 0.05, ** *p* ≤ 0.01, *** *p* ≤ 0.001, **** *p* < 0.0001).

## Data Availability

Data are available upon request.
